# Supplementary material for: Superorganismal anisogamy: queen–male dimorphism in eusocial insects
Source: Proc Biol Sci. 2020 Jun 10;287(1928):20200635. doi: 10.1098/rspb.2020.0635 (PMC7341914; doi:10.1098/rspb.2020.0635)
Supplement: Appendix: stability analysis [file rspb20200635supp1.pdf]

Appendix – stability analysis

To confirm evolutionary stability, we must ensure for each model that the candidate trait value satisfies the criterion

$$\left.\frac{\partial^2 \hat{w}_x}{\partial \hat{x}^2}\right|_{\hat{x}=x=x^*} < 0$$

and to confirm convergence stability, the criterion

$$\frac{\partial}{\partial x}\left(\left.\frac{\partial \hat{w}_x}{\partial \hat{x}}\right|_{\hat{x}=x}\right)\Bigg|_{x=x^*} < 0$$

(see e.g. [1-3]).

Model 1 stability analysis

Queen evolutionary stability:

$$\left.\frac{\partial^2 \hat{w}_x}{\partial \hat{x}^2}\right|_{\hat{x}=x=x^*} = -\frac{e^{-1}M}{(\alpha+\beta)^3}$$

Queen convergence stability:

$$\frac{\partial}{\partial x}\left(\left.\frac{\partial \hat{w}_x}{\partial \hat{x}}\right|_{\hat{x}=x}\right)\Bigg|_{x=x^*} = -\frac{e^{-1}M}{(\alpha+\beta)^3}$$

Male evolutionary stability:

$$\left.\frac{\partial^2 \hat{w}_y}{\partial \hat{x}^2}\right|_{\hat{x}=y=y^*} = -\frac{e^{-\frac{\beta+x}{x}}M}{\alpha^2 x}$$

Male convergence stability:

$$\frac{\partial}{\partial y}\left(\left.\frac{\partial \hat{w}_y}{\partial \hat{y}}\right|_{\hat{y}=y}\right)\Bigg|_{y=y^*} = -\frac{e^{-\frac{\beta+x}{x}}M}{\alpha^2 x}$$

These are all negative, and hence the model one queen and male equilibria are stable (evolutionary and convergence stability).

Model 2 stability analysis

Queen evolutionary stability:

$$\left.\frac{\partial^2 \hat{w}_x}{\partial \hat{x}^2}\right|_{\hat{x}=x=x^*} = -\frac{e^{-1}M}{\beta^3}$$

Queen convergence stability:

$$\frac{\partial}{\partial x}\left(\left.\frac{\partial \hat{w}_x}{\partial \hat{x}}\right|_{\hat{x}=x}\right)\Big|_{x=x^*} = -\frac{e^{-1}M}{\beta^3}$$

These are negative, and hence the queen equilibrium is an ESS and convergence stable, as long as $\beta > \delta$ (i.e. the queen equilibrium is larger than the minimum size).

For males we cannot use the usual derivative tests, but we can deduce stability as follows:

For evolutionary stability, consider a population at $y^* = \delta$. A mutant queen producing smaller males cannot invade, because these males would be inviable. A mutant producing larger males cannot invade either, because $\hat{n}_y$ is a decreasing function of male size in the fitness function $\hat{w}_y = \hat{n}_y \frac{n_x}{n_{y^*}} s(x)$. Hence $y^* = \delta$ is an ESS.

Similarly, it is clear that $\left.\frac{1}{w_y}\frac{\partial \hat{w}_y}{\partial \hat{y}}\right|_{\hat{y}=y} = \left(\frac{\hat{n}'_y}{n_y}\right)\Big|_{\hat{y}=y} = -\frac{1}{y}$ is negative for all values of $y^* > \delta$. Smaller sizes are inviable. Hence $y^* = \delta$ is convergence stable.

Model 3 stability analysis

Queen evolutionary stability:

$$\left.\frac{\partial^2 \hat{w}_x}{\partial \hat{x}^2}\right|_{\hat{x}=x=x^*} = \frac{b(-2+k)k^3 - ak^6}{(b+ak^2)^3}$$

This is slightly more complex than model 1 or 2. It is easy to see that this expression is negative when *k*=1 (this is identical to model 1 above) or *k*=2. But when *k*>2, the condition for the expression to be negative is $b(-2+k)k^3 - ak^6 < 0$, or $\frac{b}{a} < \frac{k^3}{k-2}$. In other words, the equilibrium is an ESS for colonies founded by one or two queens. But for 3 queens or more, there is an additional condition for evolutionary stability, which stipulates that *b* should not be too large relative to *a*. However, this condition is not particularly restrictive. When *k*=3, we obtain $\frac{b}{a} < 27$, and the limiting ratio increases thereafter.

Queen convergence stability is more straightforward:

$$\frac{\partial}{\partial x}\left(\left.\frac{\partial \hat{w}_x}{\partial \hat{x}}\right|_{\hat{x}=x}\right)\Big|_{x=x^*} = -\frac{e^{-\frac{k(b+ak)}{b+ak^2}} k^6 M}{(b+ak^2)^3}$$

which is always negative.

Overall, then, the queen equilibrium is convergence stable, and it is an ESS if *k*=1 or *k*=2, or if *k*>2 and $\frac{b}{a} < \frac{k^3}{k-2}$.

Male evolutionary stability:

$$\left.\frac{\partial^2 \hat{w}_y}{\partial \hat{x}^2}\right|_{\hat{x}=y=y^*} = -\frac{e^{-\frac{b+kx}{kx}} M}{a^2 x}$$

Male convergence stability:

$$\left.\frac{\partial}{\partial y}\left(\left.\frac{\partial \hat{w}_y}{\partial \hat{y}}\right|_{\hat{y}=y}\right)\right|_{y=y^*} = -\frac{e^{-\frac{b+kx}{kx}} M}{a^2 x}$$

These are both negative, and hence the male equilibrium is an ESS and convergence stable.

References for appendix

[1] Eshel, I., Motro, U. & Sansone, E. 1997 Continuous stability and evolutionary convergence. *Journal of Theoretical Biology* **185**, 333-343.
[2] Otto, S. P. & Day, T. 2007 *A biologist's guide to mathematical modeling in ecology and evolution*. Princeton, Princeton University Press.
[3] Lehtonen, J. 2018 The Price Equation, Gradient Dynamics, and Continuous Trait Game Theory. *The American Naturalist* **191**, 146-153. (DOI:10.1086/694891).
